# Comparative performance of L1- and E6/E7-targeted HPV genotyping assays based on a unified MeltArray platform

**DOI:** 10.1128/spectrum.00206-26

**Published:** 2026-05-18

**Authors:** Yuli Liu, Huihui Qu, Feng Zhang, Shuyang Gan, Yongqing Lai, Xiaoxing Zhang, Meiying Zhong, Xinyi Yang, Ye Xu, Yiqun Liao, Qingge Li

**Affiliations:** 1 State Key Laboratory of Vaccines for Infectious Diseases, Engineering Research Center of Molecular Diagnostics of the Ministry of Education, School of Public Health, Xiamen University 12466, Xiamen, China; 2 Engineering Research Center of Molecular Diagnostics of the Ministry of Education, School of Life Sciences, Xiamen University 12466, Xiamen, China; 3 Fujian Engineering Research Center of Nucleic Acid Detection Technologies, Fujian, China; 4 Xiamen Key Laboratory of Multi-Target and Automated Genetic Testing, Fujian, China; The University of Arizona, Tucson, Arizona, USA

**Keywords:** MeltArray, genotyping, E6/E7 genes, L1 gene, human papillomavirus

## Abstract

Human papillomavirus (HPV) molecular testing is central to cervical cancer screening. Commercial assays commonly target either the L1 gene or the E6/E7 oncogenes, yet comparative performance data under standardized conditions remain limited. We developed two HPV genotyping assays targeting L1 and E6/E7 regions, respectively, using the same MeltArray platform to minimize technical variability. Both assays detect 18 high-risk and potential high-risk HPV types. A total of 2,477 clinical samples (2,034 liquid-based cytology [LBC] and 413 formalin-fixed paraffin-embedded [FFPE] specimens) were analyzed. Concordance in HPV detection and genotyping was assessed using Cohen’s kappa statistics. The assays showed 98.8% overall agreement in HPV detection (Cohen’s kappa [*κ*] = 0.976), with high concordance across LBC and FFPE samples, including high-grade squamous intraepithelial lesion, cervical intraepithelial neoplasia (CIN) 2/CIN 3, and cervical cancer. Discordant genotyping results were rare and concentrated in types with differing limits of detection (e.g., HPV39, HPV52, and HPV66). No substantial detection bias favoring either target gene was observed in cancer cases. When performed under matched analytical conditions, L1 and E6/E7-based assays provide equivalent performance in HPV detection and genotyping. These findings support their interchangeable use in cervical cancer screening, with both targets demonstrating high reliability across the full spectrum of cervical lesions.

**IMPORTANCE:**

Human papillomavirus (HPV) testing is the principal strategy for cervical cancer screening, with most commercially available assays targeting either the L1 gene or the E6/E7 oncogenes. However, the comparisons of L1 and E6/E7 targets under standardized technical conditions remain limited. In this study, we developed and evaluated two parallel HPV genotyping assays targeting the L1 gene and E6/E7 genes, respectively, using the same MeltArray platform and unified reaction conditions. Analysis of a clinical cohort comprising 2,477 patients demonstrated equivalent performance between L1 and E6/E7 assays in both cervical swab and tissue samples, supporting their interchangeable use in cervical cancer screening.

## INTRODUCTION

Human papillomavirus (HPV) is the primary etiologic agent of cervical cancer, which remains the leading cause of cancer-related morbidity and mortality in women worldwide (1). Recognizing this, the World Health Organization (WHO) recommends molecular HPV testing as the principal strategy for cervical cancer screening (2). As part of its global initiative to eliminate cervical cancer by 2030, the WHO aims to ensure that 70% of women are screened with a high-performance test by age 35 and again by 45 (3). To meet this public health goal, hundreds of molecular assays have been developed (4), most of which rely on detecting either the L1 gene or the E6/E7 oncogenes of the HPV genome (5).

The L1 gene, which encodes the major capsid protein, is highly conserved across HPV genotypes and serves as the basis for widely used consensus primer sets (e.g., MY09/11 [6], GP5+/6+ [7], and SPF10 [8]). Consequently, many commercial assays, including the Cobas 4800 HPV Test (Roche) (9), the Abbott RealTime HR HPV Test (Abbott) (10), and Anyplex II HPV HR Detection (Seegene) (11), use L1 as their primary target. However, during viral integration into the host genome, a common event in cervical carcinogenesis, the L1 region is frequently disrupted or deleted, raising concerns about its sensitivity in detecting high-grade lesions or invasive cancers (12, 13).

In contrast, the E6 and E7 oncogenes are retained and actively transcribed following integration (14). These genes are central to the oncogenic process, inactivating tumor suppressor pathways and promoting malignant transformation of cervical epithelial cells (15, 16). Thus, assays targeting E6/E7 DNA or mRNA, such as the APTIMA HPV Assay (Hologic) (17), BD Onclarity (BD) (18), and Xpert HPV (Cepheid) (19), may offer improved sensitivity for identifying clinically significant infections (20). However, comparisons across assays are often confounded by differences in assay chemistry, technological platforms, and implementation procedures, making it difficult to isolate the impact of the target gene itself (19, 21–24).

To address this challenge, we developed two parallel HPV genotyping assays targeting L1 and E6/E7, respectively, both using a MeltArray detection chemistry (25–29). This design eliminates interassay variability and enables a controlled, head-to-head comparison of target gene performance. We applied both assays to a large and clinically diverse cohort of 2,477 clinical samples, including liquid-based cytology (LBC) specimens and formalin-fixed paraffin-embedded (FFPE) tissues spanning the entire spectrum of disease severity. Our goal is to provide a robust, stage-specific evaluation of L1 vs E6/E7 as molecular targets for HPV genotyping and to clarify their relative clinical utilities in cervical cancer screening.

## MATERIALS AND METHODS

### HPV assays and reference materials

Two genotyping assays were developed on the MeltArray platform for the detection of 18 HPV genotypes, including 14 high-risk types (HPV16, HPV18, HPV31, HPV33, HPV35, HPV39, HPV45, HPV51, HPV52, HPV56, HPV58, HPV59, HPV66, and HPV68) and 4 potential high-risk types (HPV26, HPV53, HPV72, and HPV82). One assay targets the L1 gene (“L1 assay”) in a single-tube reaction. The thermal cycling profiles of the L1 assay are shown in Table S1, and result interpretation of the L1 assay is shown in Table S2. The second assay targets the E6 and E7 oncogenes (“E6/E7 assay”) in two separate reactions; a sample was considered HPV positive if either the E6 or the E7 gene of a given type was detected. The thermal cycling profiles of the E6/E7 assay are shown in Table S3, and result interpretation of the E6/E7 assay is shown in Table S4. The L1 and E6/E7 assays have been translated into commercial kits. Therefore, the specific oligonucleotide sequences and detailed design parameters are proprietary and cannot be disclosed due to contractual and intellectual property restrictions.

The L1 and E6/E7 assays were developed to use identical PCR reagents and reaction conditions (with the only differences being the primers and probes required to target the respective genomic regions), were manufactured under the same production workflow, and were released under identical quality control standards. Both assays were run on the same instrument (SLAN-96 Real-Time PCR System; Zeesan, Xiamen, China), and all outputs were interpreted using the same manufacturer software with predefined calling rules. Together, these measures reduce variability arising from platform, reagent formulation, manufacturing, workflow, and result calling, thereby supporting a controlled head-to-head comparison focused on the impact of the target region (L1 vs E6/E7).

Analytical performance, including limit of detection (LOD), specificity, and reproducibility, was evaluated using the 2nd National Reference Panel for HPV Complete Genome Genotyping (National Institutes for Food and Drug Control, China). This panel includes 23 standardized HPV plasmids: 14 high-risk types, 4 potential high-risk types, and 5 low-risk types (HPV6, HPV11, HPV42, HPV43, and HPV81) to assess cross-reactivity.

LODs for the L1 assay and the E6/E7 assay were determined using quantified plasmid constructs representing 18 high-risk and potential high-risk HPV types. For each type, 10 μL of plasmid was tested at three serial input concentrations (2, 5, and 10 copies/μL), with five replicates per concentration. The LOD was defined a priori as the lowest concentration at which 100% of replicates were positive for that HPV type.

### Analytical specificity

Specificity was assessed using plasmids representing all 23 HPV types included in our evaluation, tested at 1 × 10^5^ copies/μL (10 μL input), with three replicates per HPV type, to confirm expected type calls and absence of cross-reactivity. Reproducibility was evaluated using plasmids representing the same 18 high-risk and potential high-risk HPV types at 10 copies/μL (10 μL input), with 10 replicates per type, and results were assessed for concordance of type calls across replicates.

### Clinical specimens

A total of 2,477 clinical samples were collected between 2022 and 2024 from three institutions in China: the Second Affiliated Hospital of Fujian University of Traditional Chinese Medicine, Fujian Maternal and Child Health Hospital, and Xiamen Maternal and Child Health Hospital. The cohort included 2,034 cervical swab samples preserved in LBC medium and 413 cervical tissue samples preserved as FFPE blocks. Participants were women aged 22–65 years, non-pregnant, and without vaginal medication treatment in the preceding 3 weeks. All specimens were irreversibly de-identified prior to analysis, and access to linked medical records was not permitted under the approved ethics protocol.

Cytological classifications for LBC samples followed the Bethesda system and included normal, atypical squamous cells of undetermined significance/low-grade squamous intraepithelial lesion, high-grade squamous intraepithelial lesion (HSIL)/atypical squamous cells-cannot exclude HSIL (ASC-H), and invasive cervical cancer. FFPE tissue specimens were histologically categorized as cervical intraepithelial neoplasia (CIN) 1, CIN 2, CIN 3, or invasive cervical cancer, based on standard pathological evaluation by certified pathologists at each institution.

### Comparative testing of L1 and E6/E7 assays

HPV DNA was extracted from LBC samples using the HPV DNA Isolation Kit (Zeesan) and from FFPE specimens using the FFPE DNA Isolation Kit (Zeesan), following the manufacturer’s protocols. All DNA extracts were tested in parallel with both the L1 assay and E6/E7 assay on the SLAN Real-Time PCR System (Zeesan).

Comparison of the two assays included overall HPV detection, partial genotyping (HPV16, HPV18, and pooled “other” types), and complete genotyping for all 18 HPV types. Statistical comparisons were performed for interassay agreement using Cohen’s kappa (*κ*) statistic, interpreted as 0.00–0.20 (poor), 0.21–0.40 (fair), 0.41–0.60 (moderate), 0.61–0.80 (good), and 0.81–1.00 (excellent). All discordant results were further analyzed by Sanger sequencing using type-specific primers targeting both L1 and E6/E7 regions to determine concordance with either assay.

## RESULTS

### Analytical performance of L1 and E6/E7 assays using a National Reference Panel

Both the L1-based and E6/E7-based HPV genotyping assays were developed using MeltArray chemistry and optimized to achieve comparable analytical sensitivity and specificity. Evaluation using the 2nd National Reference Panel for the Complete Genome of Human Papillomavirus revealed limits of detection (LOD) ranging from 2 to 5 copies/μL across the 18 targeted HPV types. Specifically, the L1 assay showed slightly superior sensitivity for HPV39, HPV52, and HPV66 (LOD: two copies/μL) compared to the E6/E7 assay (LOD: five copies/μL), while performance for the remaining 15 HPV types was equivalent between the assays (Table S5).

Both assays demonstrated 100% analytical specificity, with no cross-reactivity among the 23 HPV plasmid types included in the reference panel. In repeatability testing, all 18 HPV types tested at 10 copies/μL yielded coefficient of variation (CV) values for melting temperature (Tm) below 0.3% across 10 replicates, indicating high intra-assay precision. These evaluations were independently verified by the Fujian Institute for Food and Drug Quality Control.

### Concordance of L1 and E6/E7 assays for HPV detection

**Fig 1:**
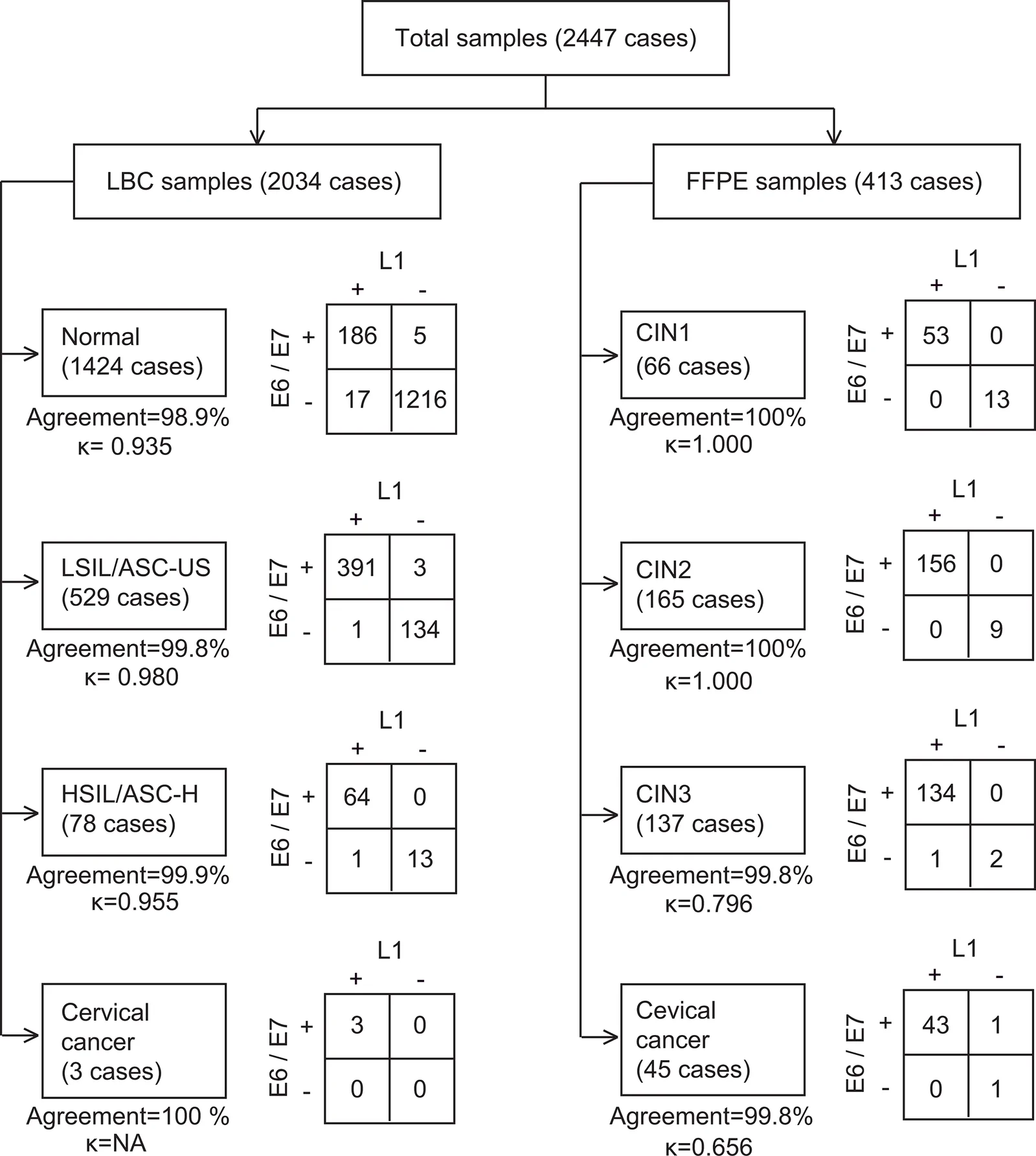
Overview of sample distribution and HPV detection concordance by lesion category using L1 and E6/E7 assays. Lesion classification for liquid-based cytology (LBC) samples was based on cytological assessment, with cervical cancer cases confirmed by colposcopy and histopathology. For formalin-fixed paraffin-embedded (FFPE) tissue samples, lesion status was determined by biopsy. Cross-tabulations of HPV detection results obtained from the L1 and E6/E7 assays are shown for each lesion category. Concordance between assays was evaluated using Cohen’s kappa (*κ*) statistic to quantify interassay agreement.

Among the 2,447 clinical samples tested (2,034 LBC samples and 413 FFPE tissue samples), the overall HPV detection agreement between the two assays was 98.8% (*κ* = 0.976). Stratified results showed 98.7% concordance in LBC samples (*κ* = 0.970) and 99.5% in FFPE samples (*κ* = 0.959). Sample distribution and HPV detection concordance by lesion category using L1 and E6/E7 assays are detailed in Fig. 1. Notably, high agreement was also observed in high-grade lesions: 98.8% in LBC samples with combined HSIL/ASC-H and cervical cancer (*κ* = 0.956) and 99.4% in FFPE samples with CIN2+, including cancer (*κ* = 0.920).

### Comparative analysis for HPV16, HPV18, and other high-risk genotypes

Targeted genotyping for HPV16, HPV18, and a pooled group of the other 16 high-risk HPV types was performed to assess assay agreement in clinically relevant genotypes. In LBC samples, the assays showed high agreement: HPV16, 99.8% concordance (*κ* = 0.981); HPV18, 99.8% (*κ* = 0.916); and other 16 HPV types, 98.9% (*κ* = 0.960). In FFPE samples, concordance remained high: HPV16, 99.3% (*κ* = 0.980); HPV18, 99.5% (*κ* = 0.931); and other types: 99.5% (*κ* = 0.989).

A total of 31 discordant LBC cases were observed in partial genotyping (Table 1). Notably, 87.1% of these occurred in samples with normal or low-grade cytology. No discordant results were observed in LBC samples classified as cervical cancer. In the FFPE group (Table 2), seven discordant results were identified in CIN2+ samples. In the LBC and FFPE discordant cases, five involved HPV16 or HPV18, of which only one was confirmed by Sanger sequencing (Table S6, sample #N388).

**TABLE 1:** Comparative analysis of HPV partial genotyping by L1 assay and E6/E7 assay for LBC samples^*a*^

HPV type	E6/E7	Overall, *n* = 2,034			Normal, *n* = 1.424			LSIL/ASC-US, *n* = 529			HSIL/ASC-H, *n* = 78			Cancer, *n* = 3		
		L1		*κ*	L1		*κ*	L1		*κ*	L1		*κ*	L1		*k*
		+	−		+	−		+	−		+	−		+	−	
Any HPV type	+	644	8	0.970	186	5	0.935	391	3	*0.980*	64	0	0.955	3	0	NA^*b*^
	-	19	1,363		17	1216		1	134		1	13		0	0	
HPV16	+	110	1	0.981	29	1	0.983	49	0	0.978	31	0	0.970	1	0	1.000
	-	3	1,920		0	1,394		2	478		1	36		0	2	
HPV18	+	22	1	0.916	4	0	0.889	16	1	0.969	2	0	0.655	0	0	NA
	-	3	2,088		1	1,419		0	512		2	74		0	3	
Pool of other 16 HPV types	+	337	7	0.960	143	4	0.934	169	2	0.981	23	1	0.970	2	0	1.000
	-	16	1,684		14	1,263		2	356		0	54		0	1	

^*a*^ ASC-US, atypical squamous cells of undetermined significance; LSIL, low-grade squamous intraepithelial lesion.

^*b*^ NA, the kappa value can’t be caculated when the consistent results between two assays (+/+ or -/-) is zero.

**TABLE 2:** Comparative analysis of HPV partial genotyping by L1 assay and E6/E7 assay for FFPE samples

HPV type	E6/E7	Overall, *n* = 413			CIN1, *n* = 66			CIN2, *n* = 165			CIN3, *n* = 137			Cancer, *n* = 45		
		L1		*κ*	L1		*κ*	L1		*κ*	L1		*κ*	L1		*κ*
		+	−		+	−		+	−		+	−		+	−	
Any HPV type	+	386	1	0.959	53	0	1.000	156	0	1.000	134	0	0.796	43	1	0.656
	-	1	25		0	13		0	9		1	2		0	1	
HPV16	+	171	1	0.980	6	0	1.000	55	0	0.978	77	0	0.970	33	1	0.942
	-	2	238		0	60		1	109		1	58		0	11	
HPV18	+	14	2	0.931	5	0	1.000	7	1	0.930	2	1	0.796	0	0	NA
	-	0	397		0	61		0	157		0	134		0	45	
Pool of other 16 HPV types	+	285	0	0.989	54	0	1.000	154	0	0.949	69	0	0.985	8	0	1.000
	-	2	126		0	12		1	10		1	67		0	37	

### Comprehensive genotyping concordance for all 18 HPV types

**Fig 2:**
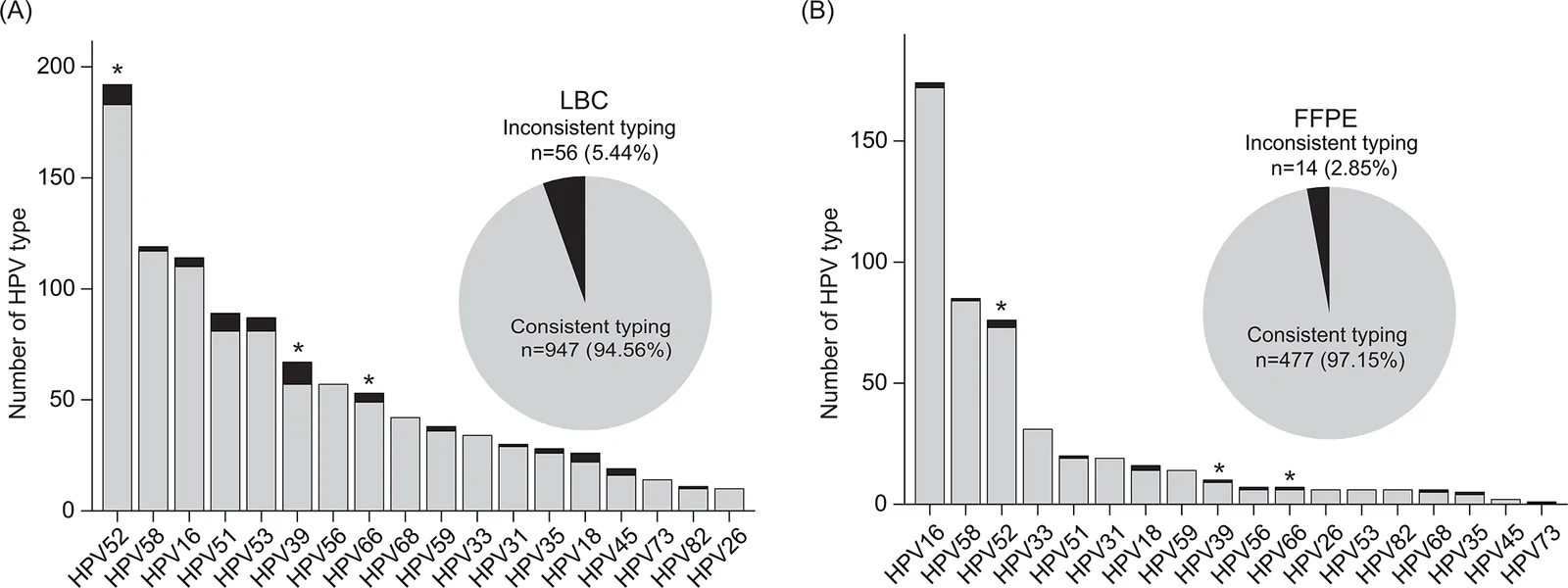
Genotyping discordance rates between L1 and E6/E7 assays by HPV type and sample type. Bar chart comparing the number of discordant genotyping calls between the L1 and E6/E7 assays across 18 HPV genotypes in (**A**) liquid-based cytology (LBC) and (**B**) formalin-fixed paraffin-embedded (FFPE) samples. Discordance was calculated as the proportion of genotype-specific calls that were non-matching between the assays. HPV types with known lower sensitivity in one assay (e.g., HPV39, HPV52, and HPV66) showed higher discordance rates. *The HPV types with inconsistent LODs between two assays.

Across the cohort, a total of 1,494 HPV genotypes were identified: 1,003 in the LBC samples and 491 in the FFPE samples. Full genotyping comparison revealed 56 discordant calls in 47 LBC samples and 14 discordant calls in 12 FFPE samples (Fig. 2). Sanger sequencing was performed on all discordant genotypes, yielding interpretable results in 21 LBC and 5 FFPE cases. Among the LBC samples, 13 results were concordant with the L1 assay, 6 with the E6/E7 assay, and 2 with neither (Table S6). In FFPE samples, three aligned with the L1 assay, one with E6/E7, and one with neither (Table S7). Notably, HPV39, HPV52, and HPV66, which had slightly lower sensitivity in the E6/E7 assay, accounted for 41.1% of discordant LBC results and 35.7% of discordant FFPE results.

## DISCUSSION

This study demonstrated a high level of concordance between L1-based and E6/E7-based HPV assays for both HPV detection and genotyping across cervical swab and FFPE tissue samples. Using a unified MeltArray platform, we found that the assays achieved an overall kappa value of 0.976, exceeding the agreement levels reported in many prior comparative studies. Importantly, the few discordant cases were primarily attributable to HPV types (e.g., HPV39, HPV52, and HPV66), for which the two assays exhibited different LODs. These findings suggest that previous discrepancies reported between assays may have stemmed more from technological variation (e.g., assay chemistry and amplification sensitivity) than from inherent differences between the L1 and E6/E7 target regions themselves.

It has been hypothesized that assays targeting E6/E7 genes may offer improved sensitivity for detecting HPV in cervical cancer specimens due to preferential retention and expression of these regions following viral genome integration. However, in our cohort, which included 48 histologically confirmed cervical cancer cases (3 LBC and 45 FFPE), only one discordant result was observed. This case tested negative by the L1 assay and positive for HPV16 by the E6/E7 assay, but subsequent type-specific sequencing failed to confirm HPV16. These findings underscore that HPV DNA targeting the L1 region is largely preserved, even in high-grade lesions and invasive cancers, and can be reliably detected when using sensitive technologies. Furthermore, missed detections in the L1 assay occurred rarely and were often within the context of multiple infections, with minimal impact on clinical interpretation.

Among the 2,034 LBC samples analyzed, 27 showed discordant HPV detection results between the assays. Notably, over 80% of these discrepancies were observed in samples classified as cytologically normal, with only a single discordant case identified in the HSIL/ASC-H group, and none in cytologically defined cancer cases. This trend remained consistent in the genotype-specific comparison (Table 1). These data suggest that while minor differences between assays may be seen in low-grade or clinically insignificant infections, both assays exhibit high sensitivity and clinical utility in detecting transforming infections and cervical cancer precursors, which are the critical targets for screening programs.

Sanger sequencing failed in 10 LBC and 2 FFPE samples with discordant results in the L1 assay, which indicated a limitation remains in this study: no single, universally accepted reference method simultaneously covering L1 and E6/E7 exists for direct head-to-head adjudication, and future studies incorporating highly sensitive sequencing-based approaches (e.g., targeted NGS) may further improve resolution in low-viral load and mixed-infection specimens.

In conclusion, this study provides robust evidence that HPV assays targeting either the L1 or E6/E7 region can achieve equivalent diagnostic performance when evaluated under a standardized and sensitive detection platform. The near-perfect agreement across a large, well-characterized sample set supports the interchangeability of these target regions in clinical genotyping, provided technical sensitivity is optimized. Both assays demonstrated excellent alignment with cytological and histological diagnoses, reinforcing their value in HPV-based cervical cancer screening. These findings offer valuable insight for assay design and may inform regulatory and clinical decision-making regarding HPV test selection in large-scale screening programs.

## SUPPLEMENTAL MATERIAL

Supplemental tables spectrum.00206-26-s0001.pdf Tables S1 to S7.

ASM does not own the copyrights to Supplemental Material that may be linked to, or accessed through, an article. The authors have granted ASM a non-exclusive, world-wide license to publish the Supplemental Material files. Please contact the corresponding author directly for reuse.
