# Supplementary material for: Comparative performance of L1- and E6/E7-targeted HPV genotyping assays based on a unified MeltArray platform
Source: Microbiol Spectr. 2026 May 18;14(7):e00206-26. doi: 10.1128/spectrum.00206-26 (PMC13340185; doi:10.1128/spectrum.00206-26)
Supplement: Supplemental tables — Tables S1 to S7. [file spectrum.00206-26-s0001.pdf]

Supplementary table 1. The PCR thermal cycling of L1 assay.

	Stage	Temperature (℃)	Time (Second)	Cycles
PCR reaction	*UNG treatment	50	120	1
	Pre-denaturation	95	180	1
	Denaturation	95	10	45
	Annealing and extension	60	35	
	Incubation	35	720	1
Melting curve analysis	Incubation	95	120	1
	Incubation	52	60	1
	Melting	Temperature increase from 52 ℃ to 92 ℃, 0.2 ℃/step		1

* UNG: Uracil DNA glycosylase

Supplementary table 2. The result interpretation for L1 assay.

HPV type	*T_m value range (℃)	Fluorescence
HPV-16	66.37~69.37	ROX
HPV-18	68.97~72.27	Quasar 705
HPV-26	73.19~76.19	ROX
HPV-31	69.69~72.69	Cy5
HPV-33	83.47~86.97	Cy5
HPV-35	81.19~84.19	HEX
HPV-39	60.47~63.77	ROX
HPV-45	74.25~77.25	Atto 425
HPV-51	58.61~61.81	FAM
HPV-52	78.43~81.93	Cy5
HPV-53	75.83~79.03	HEX
HPV-56	56.43~59.43	ROX
HPV-58	69.58~72.58	HEX
HPV-59	77.20~80.20	FAM
HPV-66	65.26~68.26	Cy5
HPV-68	64.52~67.82	FAM
HPV-73	71.40~74.40	FAM
HPV-82	84.38~87.68	ROX
β-Globin	63.48~67.68	HEX

*T_m: Melting temperature

Supplementary table 3. The PCR thermal cycling of E6/E7 assay.

	Stage	Temperature (℃)	Time (Second)	Cycles
PCR reaction	UNG treatment	50	120	1
	Pre-denaturation	95	180	1
	Denaturation	95	10	45
	Annealing and extension	55	35	
	Incubation	35	720	1
Melting curve analysis	Incubation	95	120	1
	Incubation	50	60	1
	Melting	Temperature increase from 50 ℃ to 95 ℃, 0.2 ℃/step		1

Supplementary table 4. The result interpretation for E6/E7 assay.

HPV type	T_m value range (℃)	Fluorescence
HPV-16	73.81~75.31	FAM
HPV-18	75.64~77.64	ROX
HPV-26	60.61~62.11	ROX
HPV-31	86.84~88.84	ROX
HPV-33	83.27~85.77	Atto 425
HPV-35	66.43~68.73	Quasar 705
HPV-39	58.17~60.17	HEX
HPV-45	66.32~68.32	HEX
HPV-51	75.82~78.32	HEX
HPV-52	81.02~83.52	HEX
HPV-53	59.38~61.88	Quasar 705
HPV-56	83.00~85.00	FAM
HPV-58	68.28~71.58	FAM
HPV-59	69.16~71.66	ROX
HPV-66	66.36~68.86	ROX
HPV-68	68.52~72.52	Atto 425
HPV-73	78.55~81.05	FAM
HPV-82	78.02~82.02	Atto 425
β-Globin	74.09~78.09	Quasar 705

Supplementary table 5. The LODs of L1 assay and E6/E7 assay for 18 HPV types.

HPV type	LOD of L1 assay（copies/μL）	LOD of E6/E7 assay (copies/μL)
HPV16	5	5
HPV18	5	5
HPV26	2	2
HPV31	5	5
HPV33	5	5
HPV35	5	5
*HPV39	2	5
HPV45	5	5
HPV51	5	5
*HPV52	2	5
HPV53	5	5
HPV56	2	2
HPV58	2	2
HPV59	2	2
*HPV66	2	5
HPV68	2	2
HPV73	2	2
HPV82	2	2
RPP30	5	5

* L1 assay showed slightly superior sensitivity for HPV39, HPV52, and HPV66 (LOD: 2 copies/μL) compared to the E6/E7 assay (LOD: 5 copies/μL)

Supplementary table 6. Sanger sequencing for discordant results between L1 assay and E6/E7 assay in LBC samples.

Samples	L1 assay	E6/E7 assay	Sanger sequencing	Consistent with sequencing
N131	HPV39, 52	HPV52	HPV39, 52	L1 assay
N146	HPV51, 52, 56	HPV52, 56	HPV51, 52, 56	L1 assay
N176	HPV52	Negative	HPV52	L1 assay
N332	HPV51, 53	Negative	HPV51, 53	L1 assay
N388	HPV18	Negative	HPV18	L1 assay
N428	HPV58	Negative	HPV58	L1 assay
N433	HPV52	Negative	HPV52	L1 assay
N463	HPV39, 58, 59	Negative	HPV39, 58, 59	L1 assay
6414	HPV35, 53, 56	HPV56	HPV35, 53, 56	L1 assay
6824	HPV39, 51, 53	HPV51, 53	HPV39, 51, 53	L1 assay
TP52	HPV52	Negative	HPV52	L1 assay
TP53	HPV51	Negative	HPV51	L1 assay
P170	HPV58	Negative	HPV58	L1 assay
N697	Negative	HPV66	HPV66	E6/E7 assay
N701	Negative	HPV52	HPV52	E6/E7 assay
N714	HPV35, 58	HPV35, 39, 58	HPV35, 39, 58	E6/E7 assay
6352	HPV16, 53	HPV16, 52, 53	HPV16, 52, 53	E6/E7 assay
TP62	Negative	HPV53	HPV53	E6/E7 assay
P75	HPV58	HPV58, 66	HPV58, 66	E6/E7 assay
P193	HPV68	HPV39, 53	HPV39, 68	None assay
P196	HPV53	HPV39, 51, 53	HPV39, 53	None assay

Supplementary table 7. Sanger sequencing for discordant results between L1 assay and E6/E7 assay in FFPE samples.

Samples	L1 assay	E6/E7 assay	Sanger sequencing	Consistent with sequencing
1.37	HPV26, 52, 56	HPV26, 56	HPV26, 52, 56	L1 assay
2.97	HPV16, 35	HPV16	HPV16, 35	L1 assay
3.42	HPV16, 51	HPV16	HPV16, 51	L1 assay
1.66	HPV33	HPV33, 58	HPV33, 58	E6/E7 assay
5.70	HPV18, 58	HPV58, 73	HPV18, 58, 73	None assay
